# Contribution of nursing research to fighting against COVID-19 pandemic. A systematic review*

**DOI:** 10.15649/cuidarte.2545

**Published:** 2022-10-18

**Authors:** Lyda Z. Rojas, Juliana Alexandra Hernández Vargas, Silvia Juliana Trujillo-Cáceres, Sandra Lucrecia Romero Guevara

**Affiliations:** 1 . Research Group and Development of Nursing Knowledge (GIDCEN- FCV), Research Center, Cardiovascular Foundation of Colombia. Colombia. Email: lydarojas@fcv.org Research Center Cardiovascular Foundation of Colombia Colombia lydarojas@fcv.org; 2 . High-Cost Account, High-Cost Diseases Fund, Bogotá, Colombia. Email: jhernandez@cuentadealtocosto.org High-Cost Diseases Fund Bogotá Colombia jhernandez@cuentadealtocosto.org; 3 . High-Cost Account, High-Cost Diseases Fund, Bogotá, Colombia. Email: strujillo@cuentadealtocosto.org High-Cost Diseases Fund Bogotá Colombia strujillo@cuentadealtocosto.org; 4 . GRINFER Group, Universidad industrial de Santander, School of Nursing. Colombia. Email: salurome@uis.edu.co Universidad Industrial de Santander Universidad industrial de Santander School of Nursing Colombia salurome@uis.edu.co

**Keywords:** Coronavirus Infections, Betacoronavirus, Nursing Care, Pandemics, Systematic Review, Infecciones por Coronavirus, Betacoronavirus, Atención de Enfermería, Pandemias, Revisión Sistemática, Infectes por Coronavirus, Betacoronavirus, Cuidados de Enfermagem, Pandemias, Revisao sistemática

## Abstract

**Introduction:**

The 2019 coronavirus disease (COVID-19) pandemic, should be an opportunity to ensure greater visibility of nursing in health systems and society worldwide.

**Objective:**

Review and synthesize the patterns on COVID-19 and nursing research, identifying the main journals, country of origin, language, topics, designs, and area of applicability of the results.

**Materials and Methods:**

Systematic review. Searches in PubMed, CINAHL, LILACS, and EMBASE databases (from the inception of the pandemic to May 15, 2020) were performed. Articles of any language related were related to SARS-CoV-2 infection or COVID-19 disease and nursing in any of its roles (care, management, education, among others) and using any epidemiological design or a scientific report were included. Two reviewers independently selected the studies and extracted the data. The main findings from the included studies were summarized through narrative synthesis and descriptive tables. The characteristics of the studies were presented as absolute values and proportions.

**Results:**

Three hundred and sixty-five articles were assessed for eligibility. Thirty-eight were included, published in 28 journals. Of those, 53.57% (n=15) were nursing specific. Most articles were “narrative reviews”, accounting for 23.68% (n=9). Most studies were conducted in China (n=18, 47.37%), followed by the United Kingdom and the United States. Thirty-four (89.47%) articles were published in English, followed by Portuguese and Chinese. We identified five areas of application of the results, and the most frequent was the “clinical” setting with 47.00% (n=18).

**Discussion:**

These findings are crucial to give visibility to nursing work during the emergency of the COVID-19 pandemic. Mental health was the main research topic, while the clinical setting concentrates the major number of articles. This pattern was aligned with the challenges of the initial phase of the pandemic.

**Conclusion:**

Future research should explore the current state of evidence in the main topics identified in this review and continue to give visibility to work carried out by nursing in the emergency of the COVID-19 pandemic.

## Resumen

**Introducción:**

La pandemia de la enfermedad de coronavirus 2019 (COVID-19), debería ser una oportunidad para asegurar una mayor visibilidad de la enfermería en los sistemas de salud y la sociedad en todo el mundo.

**Objetivo:**

revisar y sintetizar los patrones de investigación en enfermería y COVID-19, identificando las principales revistas, país de origen, idioma, temas, diseños y área de aplicabilidad de los resultados.

**Materiales y Métodos:**

Una revisión sistemática. Se realizaron búsquedas en las bases de datos PubMed, CINAHL, LILACS y EMBASE (desde el inicio de la pandemia hasta el 15 de mayo de 2020). Se incluyeron artículos de cualquier idioma relacionados con la infección por SARS-CoV-2 o COVID-19 y enfermería en cualquiera de sus roles (cuidado, administración, educación, entre otros) que utilizaron cualquier diseño epidemiológico o informe científico. Dos revisores seleccionaron de forma independiente los estudios y extrajeron los datos. Los principales hallazgos de los estudios incluidos se resumieron mediante una síntesis narrativa y tablas descriptivas. Las características de los estudios se presentaron como valores absolutos y proporciones.

**Resultados:**

En 325 artículos se evaluaron los criterios de elegibilidad y se incluyeron 38 publicados en 28 revistas. De ellos, el 53,57% (n=15) eran específicos de enfermería. La mayoría de los artículos fueron “revisiones narrativas”, que representan el 23,68% (n=9). La mayoría de los estudios se realizaron en China (n=18, 47,37%), seguido de Reino Unido y Estados Unidos. Treinta y cuatro (89,47%) artículos se publicaron en inglés, seguidos de portugués y chino. Identificamos cinco áreas de aplicación de los resultados y la más frecuente fue el ámbito “clínico” con un 47,00% (n=18).

**Discusión:**

Estos hallazgos son cruciales para dar visibilidad al trabajo de enfermería en la emergencia de la pandemia COVID-19. Se destaca que la salud mental fue el principal tópico de investigación mientras que el escenario clínico concentró el mayor número de artículo. Este patrón estuvo alineado con los desafíos impuestos por la fase inicial de la pandemia.

**Conclusión:**

Las investigaciones futuras deberán explorar el estado actual de la evidencia en los principales temas identificados en esta revisión y continuar dando visibilidad al trabajo realizado por enfermería en la emergencia de la pandemia por COVID-19.

## Resumo

**Introdujo:**

A pandemia da doenca coronavirus (COVID-19) de 2019 deve ser uma oportu-nidade para garantir maior visibilidade da enfermagem nos sistemas de saúde e na sociedade em todo o mundo.

**Objetivo:**

revisar e sintetizar os padróes de pesquisa em enfermagem e COVID-19, identificando as principais revistas, país de origem, língua, tópicos, desenhos e área de aplicabilidade dos resultados.

**Materiais e Métodos:**

Uma revisao sistemática. As ba-ses de dados PubMed, CINAHL, LILACS e EMBASE foram pesquisadas (Do inicio da pan-demia até 15 de maio de 2020). Artigos de qualquer lingua relacionados a infecto por SARS-CoV-2 ou COVID-19 e enfermagem foram incluidos em qualquer das suas funcóes (assistén-cia, administrado, educado, entre outras) que utilizassem qualquer desenho epidemiológico ou relatório científico. Dois revisores selecionaram independentemente estudos e extrairam os dados. As principais conclusóes dos estudos incluidos foram resumidas através de uma sintese narrativa e tabelas descritivas. As características dos estudos foram apresentadas em valores absolutos e proposes.

**Resultados:**

Em 325 artigos foram avaliados os critérios de elegibili-dade e incluidos 38 publicados em 28 revistas. Destes, 53,57% (n=15) eram especificos da enfermagem. A maioria dos artigos eram "revisóes narrativas", representando 23,68% (n=9). A maioria dos estudos foi realizada na China (n=18, 47,37%), seguida do Reino Unido e dos Estados Unidos. Trinta e quatro (89,47%) artigos foram publicados em ingles, seguidos de portugués e chines. Identificamos cinco áreas de aplicado dos resultados e a mais frequente foi a área "clinica" com 47,00% (n=l8).

**Discussao:**

Estas conclusóes sao cruciais para dar vi-sibilidade ao trabalho de enfermagem durante a emergéncia da pandemia da COVID-19. A saúde mental foi o principal tema de investigado, enquanto que o cenário clinico concentra o maior número de artigos. Este padrao foi alinhado com os desafios da fase inicial da pande-mia.

**Conclusao:**

A investigado futura deve explorar o estado atual das provas nos principais tópicos identificados nesta revisao e continuar a dar visibilidade ao trabalho realizado pela enfermagem na emergéncia da pandemia da COVID-19.

## Introduction

The outbreak of coronavirus disease 2019 (COVID-19) caused by the new SARS-CoV-2 coronavirus was first reported on December 31, 2019, in Wuhan, China, and has been spread to 188 countries becoming in a pandemic^1^. As of June 29, 2020, a total of 10.199.798 cases and 502.947 deaths have been confirmed worldwide. COVID-19 is characterized by clinical manifestations such as fever, non-productive cough, dyspnea, myalgia, fatigue, normal or decreased white blood cell counts, radiographic evidence of pneumonia, organ dysfunction (shock, acute respiratory distress syndrome- ARDS, acute heart or acute kidney damage) and death can also occur in severe forms of the disease^2^. However, other manifestations such as headache or abdominal pain, diarrhea, loss of taste, or smell have been added to the clinical spectrum, during the course of the pandemic^3^. Public health emergencies such as those faced with COVID-19 require accurate and real-time information to guide effective responses^4^.

A part of the timely response is led by nursing professionals who, not only from the clinical area but also from management, epidemiological analysis, and public health carry out interventions to reduce the individual and collective impact of the pandemic. However, there is concern about the invisible work performed by nursing staff, who have been called the “silent heroes”. This can be partially explained by the 19th-century Christian concept of self-denial which is still present in the profession. Additionally, nursing is still considered an almost natural "female job" and probably due to its social devaluation, characterized by gender hierarchies, the performance of professional nurses in the fight against epidemics and pandemics has been poorly documented^5^.

Officially, 2020 has been declared the “International Year of the Nurse and Midwife” by the World Health Organization (WHO) and it's also with the bicentennial of the birth of the nursing mother, Florence Nightingale and the “Nursing Now” campaign, which pretend to generate global awareness of the nurses' value. The above, added to the COVID-19 pandemic, should be a powerful combination of forces to ensure greater visibility of nursing in health systems and society worldwide^6^. Therefore, this study aimed to review and synthesize the trends on COVID-19 and nursing research, identifying the main journals, country of origin, language, topics, type of design, and area of applicability of the results.

## Material and Methods

### Data sources and search strategy

This review was conducted following a recently published guide on how to conduct a systematic review and was reported according to PRISMA guidelines^7^^,^^8^. An extensive search for publications until May 15, 2020, was performed in four electronic databases (PubMed, CINAHL, LILACS, and EMBASE), without language restriction. The search was quite sensitive and combined the terms MeSH [COVID-19] and [Nursing]. Search details are described below: PubMed ("nursing"[Subheading] OR "nursing"[All Fields] OR "nursing"[MeSH Terms] OR "nursing"[All Fields]) AND ("COVID-19"[All Fields] OR "COVID- 2019"[All Fields] OR "severe acute respiratory syndrome coronavirus 2"[Supplementary Concept] OR "severe acute respiratory syndrome coronavirus 2"[All Fields] OR "2019-nCoV"[All Fields] OR "SARS-CoV- 2"[All Fields] OR "2019nCoV"[All Fields] OR (("Wuhan"[All Fields] AND ("coronavirus"[MeSH Terms] OR "coronavirus"[All Fields])) AND (2019/12[PDAT] OR 2020[PDAT]))); CINAHL and Lilacs (COVID-19 AND Nursing) and EMBASE ('covid 19' AND nursing). In order to retrieve additional publications, we checked the reference lists of the studies included in the current review.

### Study selection and eligibility criteria

The publications were eligible if they: (i) were related to SARS-CoV-2 infection or COVID-19 disease and nursing in any of its roles (care, management, education, among others); (ii) were performed in any setting (community, clinical and other); (iii) Type of study: were conducted using any epidemiological design or a scientific report which follows the structure introduction, methods, results, and discussion or conclusions and (iv) were written in any language. News in any format were excluded. Considering these criteria, two independent reviewers evaluated the titles and abstracts of all initially identified publications. Subsequently, articles included in the first phase were reviewed in full text, and those who met the inclusion criteria were included. Any disagreement was resolved by consensus or consultation with a third independent reviewer.

### Quality of evidence assessment

The quality of the included studies was not evaluated since we are not intended to conclude a specific topic, and the main purpose of this review was to investigate the trend of nursing and COVID-19 research using both qualitatively and quantitatively approaches.

### Data extraction and statistical analysis

From each article, common elements of data were extracted using an Excel document prepared by one of the authors, including first author, journal (name, DOI, journal category ranking, H index), country of origin, language, main topic, design, title, objective, area of application of the results, study population, and sample size. During this process, two reviewers independently extracted the information. In case of disagreement, the decision was made by consensus and, if necessary, a third reviewer was consulted. Then, data were exported into Stata version 15 for further analysis. The main findings from the included studies were summarized through narrative synthesis and descriptive tables. The characteristics of the studies were presented as absolute values and proportions. A data-set has been developed with the reported findings of the submitted manuscript with public access in Mendeley^9^.

## Results

**Figure 1:**
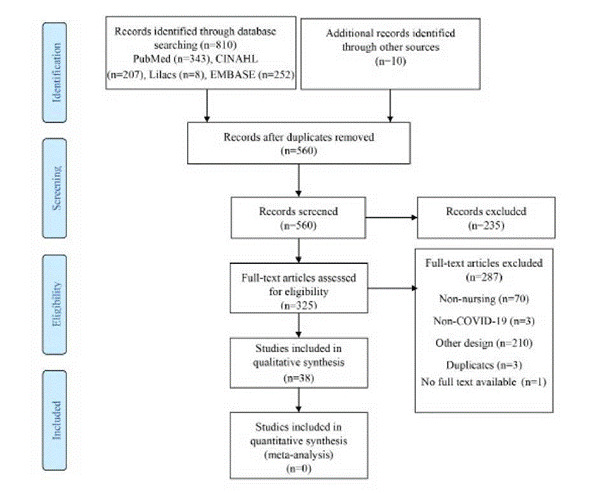
Flow chart of the articles included in the systematic review

We identified 560 citations and after a title and abstracts screening, 325 articles were selected for a detailed full text evaluation. Of these, 38 articles met our inclusion criteria and were included in the review (Figure 1).

**Table 1:** The journals of nursing identified in this systematic review (n=15)

Recently, 28 journals have published research in the coronavirus and nursing field, and 15 journals (53.57%) were nursing. Four journals contained 31.57% of nursing publications: International Journal of Nursing Sciences (n=6, 15.9%), Revista Cogitare Enfermagem (n=2, 5.26%), British Journal of Community Nursing (n=2, 5.26%) and Journal of Pain and Symptom Management (n=2, 5.26%). Table 1 shows the nursing journals identified in the review, their subject category, and ranking in the 2018 SCImago Journal Ranking (SJR) as well as their SJR and H index. The most common topics were “Nursing” (n=7, Four Q1, Two Q2 and, one Q4), followed by “Critical care Nursing, Q1”, “Medical and Surgical Nursing, Q1”, “Emergency Nursing, Q2” and “Advance and Specialized Nursing, Q3”. The highest h-index was for the Journal of Pain and Symptom Management (h-index=129), followed by the Journal of Clinical Nursing (h-index=87), Critical Care Nursing (h-index=51), Emergency Nursing (h-index=43), and Nurse Education in Practice (h-index=39) (Table 1). Journal	n (%) article (s)	Journal category (ranking) 2018*	SJR*	H-index*
International Journal of Nursing Sciences	6 (15.79)	Nursing (Q2)	0.30	9
Revista Cogitare Enfermagem	2 (5.26)	Nursing (Q4)	0.11	1
British Journal of Community Nursing	2 (5.26)	Community and Home Care (Q2), Medicine (Q3)	0.34	25
Journal of Pain and Symptom Management	2 (5.26)	Anesthesiology and Pain Medicine (Q1), Medicine (Q1), Nursing (Q1)	1.63	129
Chinese Nursing Research	1 (6.66)	---	--	---
Intensive & Critical Care Nursing	1 (6.66)	Critical Care Nursing (Q1)	0.54	51
Journal Emergency Nursing	1 (6.66)	Emergency Nursing (Q2)	0.33	43
Journal of Clinical Nursing	1 (6.66)	Medicine (Q2), Nursing (Q1)	0.77	87
Journal of Neuroscience Nursing	1 (6.66)	Endocrine and Autonomic Systems (Q3), Medical and Surgical Nursing (Q1), Neurology clinical (Q3), Surgery (Q3)	0.43	39
Journal of Nursing Management	1 (6.66)	Leadership and Management (Q1)	1.07	65
Journal of Nursing Regulation	1 (6.66)	Issues, Ethics and Legal Aspects (Q2), Nursing (Q2)	0.44	13
Nephrology Nursing Journal	1 (6.66)	Advanced and Specialized Nursing (Q3), Medicine (Q3), Nephrology (Q4)	0.18	31
Nurse Education in Practice	1 (6.66)	Education (Q1), Medicine (Q2), Nursing (Q1)	0.81	39
Nursing Standard	1 (6.66)	Medicine (Q4)	0.14	39
Practice Nursing	1 (6.66)	Nursing (Q1)	0.10	1

*SCImago Journal Rank (SJR). Source: Compilation based on the information of articles.

Most articles were “narrative reviews”, accounting for 23.68% of the total (n=38), followed by “cross sectional study” (n=8, 21.05%) and, “experience” (n=7, 18.42%). We also identified other types, such as “qualitative study” (n=3, 7.89%), “quasi-experimental” (n=3, 7.89%), “clinical guidelines” (n=2, 5.26%), among others. The results of the research subject provide insight into the trends, frequency of use, and distribution of topic categories. The most frequent topics were: mental health (n=10, 26.32%), management (n=7, 18.42%), prevention and training program (n=4, 10.53%), clinical care and aging (n=3, 7.89%), education and working conditions (n=2, 5.26%) with the same proportion for each category. Besides, subject as palliative care, policy and health travel were studied. There was a low geographic reach, representing 11 countries. Most studies conducted in China (n=18, 47.37%), followed by the United Kingdom and the United States with the same contributions (n=5, 13.16%), Brazil (n=3, 7.89%) and the other countries such as Australia, Canada, Italy, Japan, New Zealand, Singapore and Switzerland (n=7, 18.42%). Thirty-four (89.47%) articles were published in English followed by Portuguese and Chinese with the same proportion (n=2, 5.26%) (Table 2).

**Table 2:** Summary of the general characteristics of included studies in this systematic review (n=38)

Characteristic	n (%)
Document type	
Narrative review	9 (23.68)
Cross-sectional study	8 (21.05)
Experience	7 (18.42)
Quasi-experimental	3 (7.89)
Qualitative study	3 (7.89)
Clinical guidelines	2 (5.26)
Other (case report, series cases, experimental, management plan, reflection, systematic review)	6 (15.79)
Main topic	
Mental health	10 (26.32)
Management	7 (18.42)
Prevention	4 (10.53)
Training program	4 (10.53)
Clinical care	3 (7.89)
Care of the older people	3 (7.89)
Education	2 (5.26)
Working conditions	2 (5.26)
Other (palliative care, policy and travel health)	3 (7.89)
Country of origin	
China	18 (47.37)
United Kingdom	5 (13.16)
United States	5 (13.16)
Brazil	3 (7.89)
Other (Australia, Canada, Italy, Japan, New Zealand, Singapore and	7 (18.42)
Switzerland)	
Language published	
English	34 (89.47)
Portuguese	2 (5.26)
Chinese	2 (5.26)

Source: Compilation based on the information of article

**Figure 2:**
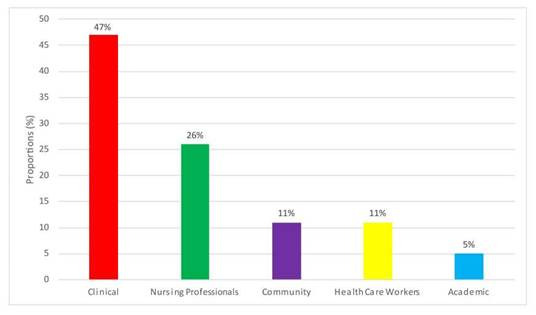
Area of application of the results found in this review

Source: Compilation based on the information of articles

We identified five areas of application of the results in this review. The most frequent was “clinical” setting with 47.0% in research topics as management, prevention, clinical and palliative care, following by the area “Nursing Professionals” with 26.0% mainly studied mental health issues, “Health Care Workers” area with 11.0% also mostly focused on mental health, “Community” area with 11.0% including aging care and travel health topics, and finally “Academic” area with 5.0% (Figure 2). The general characteristics of the included articles are shown in Table 3.

**Table 3:** General characteristics of published articles that studied COVID-19 and nursing (n=38)

First Autor	Country	Language	Main Topic	Design	Objective	Application Area	Population	Sample Size
Arons M et al.^10^	United States	English	Care of the older people	Cross-sectional	To assess the transmission and identify infections of residents with symptom-based screening.	Community	Older people residents skilled nursing facility in King Country Washington	89
Baker E et al.^11^	United Kingdom	English	Care of the older people	Narrative review	Propose a holistic model to guide community health professionals easily adapted to the COVID-19 pandemic.	Community	NA	NA
Bezerra I.^12^	Brazil	Portuguese	Education	Narrative review	To describe the challenges of using remote technologies in nursing education during the COVID-19 pandemic.	Academic	NA	NA
Fusi-Schmidhauser T et al.^13^	Switzerland	English	Palliative care	Management plan	Describe the plan of care for patients who are not candidates for ventilation due to COVID-19 disease.	Clinical	NA	NA
Hammerschmidt KS et al.^15^	Brazil	Portuguese	Care of the older people	Narrative review	Reflect on health aspects in the elderly during the COVID-19 pandemic.	Community	NA	NA
Harwood L.^16^	Canada	English	Management	Narrative review	To offer points to nephrology nurses in their approach to providing an essential service during pandemics.	Nursing professionals	NA	NA
Hillier MD.^17^	United Kingdom	English	Prevention	Narrative review	To be up-to-date to the nurses in the evidence based guidelines to the correct effective hand hygiene and its compliance.	Clinical	NA	NA
Jin Y et al.^18^	China	English	Management	Clinical guidelines	To develop an up-to-date guideline for frontline clinicians and public healthcare professionals that managing 2019- nCoV pandemics.	Clinical	NA	NA
Gawthrop M.^14^	United Kingdom	English	Travel health	Narrative review	Up-to-date	Community	NA	NA
Jowseay T et al. ^19^	New Zealand	English	Education	Systematic review	To identify approaches to blended learning and distance education for pre-registration nursing students.	Academic	NA	NA
Kang L et al.^20^	China	English	Mental health	Cross-sectional	To evaluate the sources of acute stress among women health workers and the immediate psychological impact.	Health care workers	Nurses and physicians working in Wuhan	994 (183 Physicians and 811 Nurses)
Li G et al. ^21^	China	English	Mental health	Cross-sectional	To implement Kirkpatrick's model in a nurse training program and reduce cases of infection in medical staff during the COVID-19 epidemic.	Health care workers	Doctors, nurses and medical technicians women in all clinical departments of Tongji Hospital	4369
Li Z BS et al.^22^	China	English	Training program	Quasi- experimental	To implement Kirkpatrick's model in a nurse training program and reduce cases of infection in medical staff during the COVID-19 epidemic.	Clinical	Emergency surgery department nursing staff, Tongji Hospital	35
Li Z et al.^23^	China	English	Mental health	Cross-sectional	To evaluate the vicarious traumatization and associated factors among medical staff during COVID-19.	Nursing professionals	General public (non-medical staff), front-line and non-front line nurses	214 general public and 526 nurses (234 front line and 292 non-front-line nurses)
Lippert A.^24^	United States	English	Policy	Narrative review	To describe legislation changes in nursing practice policies across United States in order to face the outbreak	Nursing professionals	NA	NA
Liu Q et al.^25^	China	English	Mental health	Qualitative study	To describe the experiences health-care providers caring in the early stages of the COVID-19 outbreak.	Health care workers	Physicians and nurses in Hubei, China	13 (9 nurses and 4 physicians)
Liu Y et al.^26^	China	English	Management	Experience	To present the emergency management of nursing resources and supplies in a general hospital to the COVID-19 outbreak.	Clinical	NA	NA
Lucchini A et al.^27^	Italy	English	Working conditions	Series cases	To describe the increase of nursing workload in intensive care units (ICUs) for COVID-19 patients	Clinical	ICU patients with and without COVID-19	15 COVID-19 patients and 474 ICU patients
Maben J et al.^28^	United Kingdom	English	Mental health	Narrative review	To discuss evidence-based guidance for addressing the needs (physical and psychological) of nurses during the COVID-19 pandemic.	Nursing professionals	NA	NA
Miranda FMA et al.^29^	Brazil	English	Working conditions	Reflexion	To reflect on the working conditions of the nursing professionals and the impact on their lives during the pandemic.	Nursing professionals	NA	NA
Mo Y et al.^30^	China	English	Mental health	Cross-sectional	To investigate the work stress and the related factors among Chinese nurses who are supporting Wuhan in fighting against COVID-19.	Nursing professionals	Nurses from Guangxi supporting Wuhan	180
Monica FPE et al.^31^	Singapore	English	Prevention	Case report	To describe the preparation and response of the nurses in Singapore General Hospital to the COVID-19 outbreak.	Clinical	NA	NA
Newby J et al.^32^	United States	English	Management	Experience	To share nursing innovations to use protective equipment and promote electronic communication to exchange knowledge and experience during the pandemic.	Clinical	NA	NA
Philips K et al.^33^	United States	English	English	Cross-sectional	To describe the implementation of the unit for attending adults with professional experts in pediatrics due to the COVID-19 pandemic.	Clinical	Adult patients with COVID-19 treated and managed by pediatric providers and nurses at the Children's Hospital of Montefiore	100
Robinson P.^34^	United Kingdom	English	Clinical care	Narrative review	To describe how SARS-CoV-2 affects people and summarize the clinical guidelines	Clinical	NA	NA
Saitoh A et al.^35^	Japan	English	Training program	Quasi- experimental	To evaluate hand hygiene adherence before attending patients and evaluate the adherence to a multimodal intervention implemented.	Health care workers	Health care workers at four tertiary hospitals in Niigata, Japan	1,630 observations pre-intervention and 1,630 post intervention
Schwedhelm M et al.^36^	United States	English	Prevention	Experience	To update a screening algorithm to identify and isolate suspected cases and their use and adaptation to address potential cases of COVID-19.	Clinical	NA	NA
Sheng X et al.^37^	China	Chinese	Mental health	Cross-sectional	To investigate the psychological status and sleep quality of nursing interns during the outbreak of COVID-19 and provide evidence for appropriate interventions.	Nursing professionals	Nursing interns	95
Soma M et al.^38^	Australia	English	Prevention	Experience	To explore a structured way to reduce aerosolization of secretions, decrease open airway time and minimize staff exposure.	Clinical	NA	NA
Song Y et al.^39^	China	English	Management	Quasi- experimental	To use reengineering theory to evaluate the intravenous infusion workflow or optimizing the process in patients with COVID-19.	Clinical	Confirmed patients with COVID-19 and nurses the Second Hospital of Nanjing	30 patients with COVID-19 30 front-line nurses
Sun N et al.^40^	China	English	Mental health	Qualitative study	To understand the subjective psychological experience of nurses and to provide fundamental data of nurses caring for COVID-19 patients.	Nursing professionals	Nurses caring for patients with COVID-19 in the First Affiliated Hospital of Henan University of Science	20
Wang H et al.^41^	China	English	Management	Experience	To summarize contingency management strategies of the Nursing Department for the attention of patients with COVID-19.	Clinical	NA	NA
Nursing Department of Tongji Hospital et al.^42^	China	English	Clinical care	Clinical guidelines	To standardize the holistic care for patients with severe COVID-19 in China.	Clinical	NA	NA
Wu L et al.^43^	China	Chinese	Training program	Experience	To explore the effect of applying the training nurses model oriented to the outpatient diagnosis of new COVID-19.	Clinical	Nurses at fever clinics and isolation rooms	300
Xu C et al.^44^	China	English	Management	Experience	To summarize the management strategies for the prevention and control of nosocomial infections and COVID-19 in nonisolated areas in a general hospital.	Clinical	NA	NA
Yifan T et al.^45^	China	English	Mental health	Cross-sectional	To analyze the symptoms and causes of somatic symptom disorder and associated risk	Nursing professionals	Nurses at COVID-19 pneumonia ICUs employed as a full-time at Jiangsu Province Hospital	140
Yin X et al.^46^	China	English	Mental health	Qualitative study	To understand the psychological needs of front line nurses attending the COVID-19 epidemic and provide a perspective for interventions.	Nursing professionals	Nurses at the front-line from a tertiary general hospital in Wuhan who had cared for patients with COVID-19	10
Zhou T et al.^47^	China	English	Training program	Experimental	To analyze the application effect of the combined mode of Massive Open Online Course during the COVID-19 epidemic in the distance	Clinical	Emergency nursing interns from Tongji Hospital Affiliated to Tongji Medical College of Huazhong University of Science and Technology	60

## Discussion

In the present review, we summarize the research patters on COVID-19 and nursing, identifying that most articles were published in the International Journal of Nursing Sciences. As expected, China was the country with the highest number of publications. Furthermore, most articles have been published in English language and narrative reviews were the most frequent type of documents, followed by cross-sectional studies, and experiences. The highlighted topics and application setting of the results, were mental health and clinical area, respectively.

Regards journals, only four nursing journals were in Q1 and two of them had h-index higher than 80. The above allows evidence of the nursing development achieved in the last years about the quality of scientific production in terms of writing, evaluating, and editing research articles^48^. Additionally, it is necessary to publish in nursing-specific journals, which allow greater visibility by nurses' staff worldwide. It is also important to highlight that English is the official language in scientific research, and that was reflected in our review, with 89.47% of articles published in this language.

On the other hand, it is important to recognize the role of countries such as China (center of the pandemic), the United States and the United Kingdom, which had the greatest contribution to nursing publications. In Latin America, most studies were performed in Brazil, where the pandemic came first, and has one of the most advanced nursing research networks in the region with the established Virtual Library on Nursing Health (BVS, by its acronym in Spanish) with the support of the Latin American and Caribbean Center in Health Information (BIREME/PAHO/WHO)^49^. Further, according to the PAHO report entitled "Doctoral training in nursing in Latin America and the Caribbean", Brazil is the country with the highest offer of nursing Ph.D. programs (n=37) and an important proportion (25%) have a long history and recognition. Also, 92% of nurses have been trained in the research field which has supported the country's leadership in nursing research^50^.

In order to analyze the type of scientific publications, it is necessary to mention that, nursing is a young profession that has mainly depended on knowledge developed by other health disciplines. Indeed, nursing training in research is recent, and also the lack of time for research activities in most professional settings has limited the type of designs used in nursing studies^51^^,^^52^. Despite undeniable progress, there are important challenges to face. Among those, the most important is the gap between nursing research and practice, especially in developing countries where the proportion of nurses with postgraduate training is low and most of them work in the education field^53^. Following the publication types, we found a huge number of editorials, reflections, and narrative reviews focused on challenges established by the pandemic. Despite, these are important, they are mainly based on personal opinions and lack of scientific methodology^52^. From included articles, one of the most frequent was the report of experiences, which has the lowest evidence level and does not allow making clinical recommendations^54^. Given the pandemic and their unexpected trends, the report of experiences has been a frequent publication useful in implementing effective actions to decrease the COVID-19 contagion and its consequences^26^^,^^32^^,^^36^^,^^38^^,^^43^.

The main type of study was the narrative review^12^^,^^14^^,^^16^^,^^17^^,^^24^^,^^28^^,^^34^, which is appropriate for the initial phase of the pandemic due to its high accessibility and easy interpretation, especially in an unprecedented acute condition with no previous scientific evidence^55^. Following the same argument, cross-sectional studies were also frequent because their main objective is to describe a health condition^56^. They are relevant to understand the patterns of COVID-19 under different conditions of public and clinical health. In this review, we identify that cross-sectional studies were used to describe the mental health status of health care professionals, especially nurses because they are in the front line of clinical attention^10^^,^^20^^,^^21^^,^^23^^,^^30^^,^^33^^,^^37^^,^^39^^).^

We also found qualitative studies, which have achieved greater visibility in research because of their usefulness in understanding human behavior and social interactions. They have also been valuable to identify the feelings and concerns of people about the pandemic in both, community and clinical areas^25^^,^^39^^,^^40^^,^^46^. Otherwise, there is a lack of experimental designs in the field of nursing and COVID-19 research, identifying only three quasi-experimental studies^22^^,^^35^^,^^39^ y one experimental^47^, mainly focused on the evaluation of educational models and adherence to preventive measures by healthcare professionals. Although they are studies with a higher level of evidence and their results can be translated into clinical recommendations, a limited number of those were expected because of their high cost and logistic^56^.

Regarding the main topics researched by nursing in the COVID-19 pandemic, mental health and management stand out. A high percentage of articles as focused on mental health and mainly researched in health care workers (HCWs)^20^^,^^21^^,^^23^^,^^25^^,^^28^^,^^37^^,^^40^^,^^45^^,^^46^ The antecedents of previous influenza A/ H1N1 and severe acute respiratory syndrome (SARS) pandemics could explain this, where it was documented that HCWs experienced high levels of stress, anxiety, and low mood. Additionally, these psychological problems in HCWs have negative consequences for organizations as the extreme pressures experienced during a pandemic may increase the risk of burnout, generating adverse outcomes at the of individual well-being, in patient care and the health system, so during the SARS outbreak, the emotions expressed by health workers were associated with resignations and poor work performance, therefore, the mental health status in HCWs has picked up interest during the COVID-19 pandemic^57^^,^^58^. In this way, a study on the factors associated with mental health outcomes in health workers in China treating patients with COVID-19 found that women, nurses, people in Wuhan and front-line workers had a high risk of developing unfavorable mental health outcomes and may need psychological support or interventions^59^. Similar results were found by Huang JZ et al. where the incidence of anxiety in female medical personnel was higher compared to men, and the incidence of anxiety in nurses was higher than that of doctors^58^.

On the other hand, nursing boards and their leaders play a key role in the management and quality of care provided to patients with COVID-19. They usually work as part of a multidisciplinary team and lead the implementation of care based on the best available evidence and promote patient and health care worker's safety. The above implies active nursing participation in new initiatives and clinical interventions according to the high healthcare demand and the new epidemiologic patterns established by COVID-19^60^. In consequence, it is important to highlight the nursing participation in the development of a rapid advice guideline is suitable for the first frontline doctors and nurses, managers of hospitals and healthcare sections, community residents, public health persons, relevant researchers, and all person who are interested in the 2019-nCoV^18^. Likewise, there were four studies where nursing staff describe their experience about human resources and supplies management, as well as, prevention and control actions in non-isolated areas, offering some usefulness advice^26^^,^^32^^,^^41^^,^^44^. Finally, we found some studies focused on considerations for nephrology nursing and the effect of optimization of intravenous infusion therapy in isolated patients with COVID-19^16^^,^^39^.

By contrast, there were a few published studies related to nursing care plans despite nursing theories have been incorporated into the habitual clinical practice^61^. Just three studies were identified in this field; one of them describe nursing care in patients with COVID-19, who were not candidates to mechanical ventilation (palliative care)^13^. In another study, twenty nursing experts from China developed a consensus about holistic care in patients with severe COVID-19, including clinical evaluation, care priorities, and 13 key points to guide the interventions^42^ and finally in the last article, the authors propose a biopsychopharmacosocial approach to evaluate the impact of social distancing and isolation in the mental health the elderly population^11^.

Another finding that is striking is the underrepresentation of the community applicability area, during the initial phase of the pandemic, despite the importance of nursing actions focused on the home and the community to control the contagion. The scarce evidence applicable to community settings is consistent with the call made by nursing associations worldwide, highlighting the importance of interventions in “wards without walls”^61^, who have lost care despite being essential to release hospital occupation, avoiding complications through individualized home care for people with mild forms of the disease. Additionally, the nursing role is essential to maintain the physical and mental health of people who remain in confinement^11^. Taking into account the above, the community health area constitutes a priority scenario to focus on scientific research and its findings to make visible and intervene in the main problems that have arisen with the pandemic.

Finally, it is important to highlight that there is coherence between the main topics studied (where health care workers mental health was the most important) and the area of application of the findings, which was mostly clinical. This response to the new challenges that nursing professionals face in clinical settings due to the pandemic, which include: the admission to hospital of a high number of critically ill patients which have increased care demands on nurses and these demands must be met by an already depleted nursing workforce. Indeed, nurses are not only experiencing an increase in the volume and intensity of their work but are having to accommodate new protocols and a very ‘new normal' which includes they have to provide end-of-life care more frequently and closer interaction with family members who cannot be at the bedside because of isolation rules^28^^,^^62^.

## Strengths and limitations

The main strength of our study is the application of a highly sensitive search and the systematic methodology used in this review, which allowed us to control or decrease selection bias, a critical assessment, and synthesis of all relevant studies on COVID-19 and nursing. However, this research study did have some limitations. First, unpublished studies were not searched and publication bias may exist. Second, aspects that do not depend on the authors, such as the rapid outdated that this review may have due to the acute nature of the event of interest (COVID-19) and for the need to generate literature on this topic that provides help in managing the pandemic. Finally, it is essential to mention that the scope of this review was limited to the initial phase of the pandemic and do not reveal the current evidence in the field.

## Conclusions

During the first phase of the pandemic, nursing research was focused on mental health topics, and the clinical area was the main setting investigated. These findings are aligned with the challenges faced during this period. It is essential to mention that nursing research was visible and valuable from the beginning of the pandemic until the current situation, contributing to understanding care phenomena typical of the pandemic. Future research should explore the current state of evidence in the main topics identified in this review and continue to give visibility to work carried out by nursing in the emergency of the COVID-19 pandemic.

## References

[1] Khalatbari-Soltani S, Cumming RG, Delpierre C, Kelly-Irving M (2020). Importance of collecting data on socioeconomic determinants from the early stage of the COVID-19 outbreak onwards. J Epidemiol Community Health.

[2] Yang X, Yu Y, Xu J, Shu H, Xia J, Liu H (2020). Clinical course and outcomes of critically ill patients with SARS-CoV-2 pneumonia in Wuhan, China: a single-centered, retrospective, observational study. Lancet Respir Med.

[3] Bolay H, Gul A, Baykan B (2020). COVID-19 is a Real Headache!. Headache.

[4] Smith MJ, Upshur REG, Emanuel EJ (2020). Publication Ethics During Public Health Emergencies Such as the COVID-19 Pandemic. Am J Public Health.

[5] Nolte K. (2020). Pandemic and Epidemic History as Nursing History?. NTM.

[6] Wood C. (2020). Nursing in a pandemic. Br J Nurs.

[7] Muka T, Glisic M, Milic J, Verhoog S, Bohlius J, Bramer W (2020). A 24-step guide on how to design, conduct, and successfully publish a systematic review and meta-analysis in medical research. Eur J Epidemiol.

[8] Moher D, Liberati A, Tetzlaff J, Altman DG, Group P (2009). Preferred reporting items for systematic reviews and meta-analyses: the PRISMA statement. PLoS Med..

[9] Rojas LZ, Hernández Vargas JA, Trujillo-Cáceres SJ, Romero Guevara SL (2022). Contribution of nursing research to fighting against COVID-19 pandemic. A systematic review. Mendeley Data.

[10] Arons MM, Hatfield KM, Reddy SC, Kimball A, James A, Jacobs JR (2020). Presymptomatic SARS- CoV-2 Infections and Transmission in a Skilled Nursing Facility. N Engl J Med..

[11] Baker E, Clark LL (2020). Biopsychopharmacosocial approach to assess impact of social distancing and isolation on mental health in older adults. Br J Community Nurs..

[12] Bezerra Pinheiro I (2020). State of the art of nursing education and the challenges to use remote technologies in the time of corona virus pandemic. J Hum Growth Dev..

[13] Fusi-Schmidhauser T, Preston NJ, Keller N, Gamondi C (2020). Conservative Management of COVID-19 Patients-Emergency Palliative Care in Action. J Pain Symptom Manage.

[14] Gawthrop M (2020). Advising Hajj and Umrah travellers in general practice. Practice Nursing.

[15] KdA Hammerschmidt, Santana R (2020). Health of the older adults in times of the COVID-19. Cogitare enferm..

[16] Harwood L. (2020). Pandemic Uncertainty: Considerations for Nephrology Nurses. Nephrol Nurs J..

[17] Hillier MD. (2020). Using effective hand hygiene practice to prevent and control infection. Nurs Stand..

[18] Jin YH, Cai L, Cheng ZS, Cheng H, Deng T, Fan YP (2020). A rapid advice guideline for the diagnosis and treatment of 2019 novel coronavirus (2019-nCoV) infected pneumonia (standard version). Mil Med Res..

[19] Jowsey T, Foster G, Cooper-Ioelu P, Jacobs S (2020). Blended learning via distance in pre-registration nursing education: A scoping review. NurseEducPract..

[20] Kang L, Ma S, Chen M, Yang J, Wang Y, Li R (2020). Impact on mental health and perceptions of psychological care among medical and nursing staff in Wuhan during the 2019 novel coronavirus disease outbreak: A cross-sectional study. Brain Behav Immun..

[21] Li G, Miao J, Wang H, Xu S, Sun W, Fan Y (2020). Psychological impact on women health workers involved in COVID-19 outbreak in Wuhan: a cross-sectional study. J Neurol Neurosurg Psychiatry.

[22] Li Z, Cheng J, Zhou T, Wang S, Huang S, Wang H (2020). Evaluating a Nurse Training Program in the Emergency Surgery Department Based on the Kirkpatrick's Model and Clinical Demand During the COVID-19 Pandemic. Telemed J E Health..

[23] Li Z, Ge J, Yang M, Feng J, Qiao M, Jiang R (2020). Vicarious traumatization in the general public, members, and non-members of medical teams aiding in COVID-19 control. Brain Behav Immun..

[24] Lippert A. (2020). Ensuring Effective and Quality Care During a Pandemic. J Nurs Regul..

[25] Liu Q, Luo D, Haase J, Guo Q, Wang X, Liu S (2020). The experiences of health-care providers during the COVID-19 crisis in China: a qualitative study. Lancet Glob Health..

[26] Liu Y, Wang H, Chen J, Zhang X, Yue X, Ke J (2020). Emergency management of nursing human resources and supplies to respond to coronavirus disease 2019 epidemic. Int J Nurs Sci..

[27] Lucchini A, Giani M, Elli S, Villa S, Rona R, Foti G (2020). Nursing Activities Score is increased in COVID-19 patients. Intensive Crit Care Nurs..

[28] Maben J, Bridges J. (2020). Covid-19: Supporting nurses' psychological and mental health. J Clin Nurs..

[29] Miranda F, LdL Santana, Pizzolato A, Saquis L (2020). Working conditions and the impact on the health of the nursing professionals in the context of covid-19. Cogitareenferm..

[30] Mo Y, Deng L, Zhang L, Lang Q, Liao C, Wang N (2020). Work stress among Chinese nurses to support Wuhan in fighting against COVID-19 epidemic. J Nurs Manag..

[31] Monica FPE, Aloweni F, Yuh AS, Ayob EBM, Ahmad NB, Lan CJ (2020). Preparation and response to COVID-19 outbreak in Singapore: A case report. Infect Dis Health..

[32] Newby JC, Mabry MC, Carlisle BA, Olson DM, Lane BE (2020). Reflections on Nursing Ingenuity During the COVID-19 Pandemic. J Neurosci Nurs..

[33] Philips K, Uong A, Buckenmyer T, Cabana M, Hsu D, Katyal C (2020). Rapid Implementation of an Adult COVID-19 Unit in a Children's Hospital. JPediatr..

[34] Robinson P. (2020). Long-term conditions and severe acute respiratory syndrome SARS-CoV-2 (COVID-19). Br J Community Nurs..

[35] Saitoh A, Sato K, Magara Y, Osaki K, Narita K, Shioiri K (2020). Improving Hand Hygiene Adherence in Healthcare Workers Before Patient Contact: A Multimodal Intervention in Four Tertiary Care Hospitals in Japan. J Hosp Med..

[36] Schwedhelm MM, Herstein JJ, Watson SM, Mead AL, Maddalena L, Liston DD (2020). Can You Catch It? Lessons Learned and Modification of ED Triage Symptom- and Travel-Screening Strategy. JEmerg Nurs..

[37] Sheng X, Liu F, Zhou J, Liao R (2020). Psychological status and sleep quality of nursing interns during the outbreak of COVID-19. Nan Fang Yi Ke Da Xue Xue Bao..

[38] Soma M, Jacobson I, Brewer J, Blondin A, Davidson G, Singham S (2020). Operative team checklist for aerosol generating procedures to minimise exposure of healthcare workers to SARS-CoV-2. Int J Pediatr Otorhinolaryngol..

[39] Song Y, Wang W, Zhang L, Sha L, Lu G (2020). Optimization of the intravenous infusion workflow in the isolation ward for patients with coronavirus disease 2019. Int J Nurs Sci..

[40] Sun N, Wei L, Shi S, Jiao D, Song R, Ma L (2020). A qualitative study on the psychological experience of caregivers of COVID-19 patients. Am J Infect Control..

[41] Wang H, Feng J, Shao L, Wei J, Wang X, Xu X (2020). Contingency management strategies of the Nursing Department in centralized rescue of patients with coronavirus disease 2019. Int J Nurs Sci..

[42] Wang H, Zeng T, Nursing Department of Tongji Hospital Affiliated to Tongji Medical College H, Nursing Department of Peking Union Medical College H, Intensive Care Professional Committee of the Chinese Nursing A, Writing Committee M (2020). Holistic care for patients with severe coronavirus disease 2019: An expert consensus. Int J Nurs Sci..

[43] Wu L, Liu L, Zhou F, Wang W, Li L, Zhang L (2020). Application of job demand-oriented training model in cultivation of support nurse moving to fever clinics and isolation ward of corona virus disease 2019 in hospital. Chinese Nursing Research..

[44] Xu C, Jin J, Song J, Yang Y, Yao M, Zhang Y (2020). Application of refined management in the prevention and control of coronavirus disease 2019 epidemic in non-isolated areas of a general hospital. Int J Nuts Sc¡..

[45] Yifan T, Ying L, Chunhong G, Jing S, Rong W, Zhenyu L (2020). Symptom Cluster of ICU Nurses Treating COVID-19 Pneumonia Patients in Wuhan, China. J Pa¡n Symptom Manage..

[46] Yin X, Zeng L (2020). A study on the psychological needs of nurses caring for patients with coronavirus disease 2019 from the perspective of the existence, relatedness, and growth theory. Int J Nurs Sc¡..

[47] Zhou T, Huang S, Cheng J, Xiao Y (2020). The Distance Teaching Practice of Combined Mode of Massive Open Online Course Micro-Video for Interns in Emergency Department During the COVID-19 Epidemic Period. Telemed J E Health..

[48] Robayo-Cruz E (2011). The Importance of Scientific Evaluation in Nursing. Aqu¡chan..

[49] Felix Lana F. (2017). Scientific communication in Nursing: Importance of netwoks in the visibility and diffusion of knowledge. Enferm Univ..

[50] Organización Panamericana de la Salud (2017). Formación doctoral en enfermería en América Latina y el Caribe. PAHO Washington, D.C..

[51] Vélez Vélez E. (2009). Research in Nursing: Basics of the Discipline. Rev Adm Sanit..

[52] Hernández-Rodríguez J, Cilleros-Pino L, Díaz-Hernández M (2018). Desarrollo de la Investigación Enfermera. Ene..

[53] Camacho Rodríguez D, Oviedo Cordoba H, Ramos de la Hoz E, Gonzalez Noguera T (2016). Bibliometric analysis of articles on nursing care published in Colombian magazines. Enf Global..

[54] Manterola C, Asenjo-Lobos C, Otzen T (2014). Hierarchy of evidence. Levels of evidence and grades of recommendation from current use. Rev Chilena Infectol..

[55] Zillmer J, Díaz-Medina B (2018). Narrative Review: elements that constitute it and its potentialities. J Nurs Health..

[56] Chrisman J, Jordan R, Davis C, Williams W (2014). Exploring evidence-based practice research. Nursing Made Incredibly Easy!. J.nurs.health..

[57] Zhang WR, Wang K, Yin L, Zhao WF, Xue Q, Peng M (2020). Mental Health and Psychosocial Problems of Medical Health Workers during the COVID-19 Epidemic in China. Psychother Psychosom..

[58] Huang JZ, Han MF, Luo TD, Ren AK, Zhou XP (2020). Mental health survey of medical staff in a tertiary infectious disease hospital for COVID-19. Zhonghua Lao Dong Wei Sheng Zhi Ye Bing Za Zhi..

[59] Lai J, Ma S, Wang Y, Cai Z, Hu J, Wei N (2020). Factors Associated With Mental Health Outcomes Among Health Care Workers Exposed to Coronavirus Disease 2019. JAMA Netw Open..

[60] Martinez Estalella G, Zabalegui A, Guerra S (2020). Management and Leadership of Nursing Services in the Emergency Plan for the Pandemic Covid-19: The Experience of the Clinic Hospital of Barcelona. Enferm Clin..

[61] Green J, Doyle C, Hayes S, Newnham W, Hill S, Zeller I (2020). COVID-19 and district and community nursing. Br J Community Nurs..

[62] Bagnasco A, Zanini M, Hayter M, Catania G, Sasso L (2020). COVID 19-A message from Italy to the global nursing community. J Adv Nurs..

